# TLE1 function and therapeutic potential in cancer

**DOI:** 10.18632/oncotarget.13278

**Published:** 2016-11-10

**Authors:** Da Yuan, Xue Yang, Zhenpeng Yuan, Yanqing Zhao, Junchao Guo

**Affiliations:** ^1^ Department of General Surgery, Peking Union Medical College Hospital, Chinese Academy of Medical Sciences and Peking Union Medical College, Beijing, China; ^2^ Institute of Hematology and Blood Diseases Hospital, Chinese Academy of Medical Sciences & Peking Union Medical College, Tianjin, China; ^3^ Department of Pediatric Cardiac Surgery, Cardiovascular Institute and Fuwai Hospital, Chinese Academy of Medical Sciences and Peking Union Medical College, Beijing, China; ^4^ Institute of Medical Information, Peking Union Medical College, Chinese Academy of Medical Sciences, Beijing, China

**Keywords:** transducin-like enhancer of split 1, tumor, pancreatic cancer

## Abstract

Groucho (Gro)/Transducin-like enhancer of split (TLE) family proteins act as co-repressors of many transcription factors, and are involved in key signaling pathways. TLE1 negatively regulates inflammation and has potential roles in various diseases, including cancer. Previous studies suggest TLE1 could be used as a diagnostic marker and is a possible therapeutic target in various malignancies. It is therefore important to elucidate the mechanisms underlying TLE1 function during cancer initiation and metastasis. In this review, we highlight the functions of TLE1 in cancer and explore targeted approaches for cancer diagnosis and treatment. In particular, we discuss the TLE1 function in pancreatic cancer.

## INTRODUCTION

Cancer is associated with high morbidity and mortality rates. It has a major impact on societies worldwide. The increasing incidence of various cancers may be explained by growth and aging of the population, as well as exposure to environmental and life style risk factors [1]. The World Health Organization (WHO) has reported 14.1 million new cancer cases and 8.2 million deaths in 2012 [2]. Importantly, pancreatic cancer is the fourth leading cause of cancer death in United States [3] and is projected to be the second leading cause of cancer death in the United States in 2030 [4]. In China, the pancreatic cancer mortality rate ranked sixth among all cancers in 2012 [5]. Over time, this rate is expected to approach those of Western countries. Current therapies for pancreatic cancer including surgical resection, radiation, and chemotherapy have not improved patient survival times and prognosis. Currently, the median survival time is only 8months and the 5-year overall survival (OS) rate is approximately 8% [3]. Therefore, a better understanding of the molecular mechanisms and pathologic processes responsible for pancreatic cancer initiation and metastasis is required in order to identify novel therapeutic strategies and improve patient survival times/quality of life.

Genomic analysis has indicated that 12 core signaling pathways are altered in 67%-100% of human pancreatic cancers [6]. Several of these are extracellular matrix signaling pathways associated with invasion and metastasis. Therefore, more detailed molecular studies are required to identify novel therapeutic targets for pancreatic cancer treatment. Groucho/Transducin-Like Enhancer of split (Gro/TLE) family proteins act as co-repressors for many transcription factors and have key roles during development. They also contribute to the pathogenesis of several cancers. Gro/TLE family proteins are effectors of the Notch, Wnt, and NF-κB signaling pathways that determine cell fate [7–9]. The family has an ortholog in *Drosophila* (Gro), which is a member of the Notch signaling pathway, and four orthologs in humans (TLE1-4) and mouse (Grg1-4). Although these proteins cannot bind to DNA individually, they can be tethered to specific regions of DNA through their association with transcription factors (e.g. Hes, Runx, and Nkx) and are potential targets for anti-cancer drugs [10]. Additionally, recent data have suggested an epigenetic basis for Groucho/Grg/TLE-mediated gene silencing [11].

In this review, we discuss the roles of TLE family proteins in cancer. We focus on the mechanisms underlying the functions of TLE1 family proteins in pancreatic cancer.

## MECHANISMS UNDERLYING TLE PROTEIN FUNCTION

TLE1-4 were first characterized as human Gro-associated proteins important for cellular differentiation [12]. TLE proteins consist of five conserved domains: Q, GP, CcN, SP, and WD40 [13]. The Q and WD40 domains are required for TLE1 function. The other domains are less conserved and regulate the subcellular localization, phosphorylationstate, and transcriptional repression activity of TLE1. TLE does not bind directly to DNA. However, as a transcriptional co-repressor, it associates with transcription factors that bind to DNA and affects the histone H3 framework to modify chromatin architecture and regulate gene expression [14]. Interestingly, TLE proteins can down-regulate the expression of transcriptional activators as well as enhance the effects of transcriptional repressors. In addition, they can convert transcriptional activators into repressors [15–17]. TLE proteins participate in osteogenesis, hematopoiesis, myogenesis, nervous system development, as well aspituitary, placenta, and intestine function either directly or indirectly [13, 18]. Thus, they have critical functions in growth and development. Recently, many studies have focused on the roles of TLE proteins (TLE1 in particular) in apoptosis and malignant progression.

## FUNCTIONS OF TLE1 IN CANCER

TLE1 is involved in diverse signaling pathways and has important roles in neurogenesis, sex determination, and segmentation during development. It associated indirectly with chromatin through binding to the N-terminal tail of histone H3 and RNA polymerase II (RNAP II) [19, 20]. TLE1 forms a homotetramer, which is mediated by the N-terminal region and is required for activation. TLEs can regulate the expression of genes that are targets of transcriptional activators, and can enhance the effects of transcriptional repressors. They can also covert transcriptional activators into repressors [17]. TLE1 is a major counter-regulator of inflammation and has potential functions in a variety of inflammatory diseases in addition to cancer [8]. TLE1 negatively regulates apoptosis and can protect caspase-independent malignant cancer cells from Bit1-induced cell death [21]. Based on these data, TLE1 has clear functions in carcinogenesis, invasion, and metastasis. In the following sections, we highlight the functions of TLE1 in various cancers. We focus on the roles of TLE1 in pancreatic cancer and explore targeted approaches for the treatment of this disease.

## TLE1 AND SYNOVIAL SARCOMA

TLE1 is expressed in synovial sarcomas. However, it is seldom detected in other soft tissue tumors. It is also not expressed in normal stromal tissue. TLE1 is therefore a robust diagnostic immunohistochemical marker for synovial sarcoma diagnosis and is a potential therapeutic target [22–25]. Recently, TLE1 was validated as a novel immunohistochemical marker. The rate of immunopositivity is greater than 80% [23, 26]. It is used in biopsies to support the diagnosis [27]. Several studies have explored the mechanisms underlying TLE1 function in synovial sarcoma. For example, Seo et al. found that inhibition of TLE1 altered cancer cell proliferation and apoptosis through suppression of Bcl-2 expression [28]. Moreover, knockdown of TLE1 in fibroblasts and synovial cells did not enhance the cytotoxicity of doxorubicin. Therefore, although the “gold standard” for synovial sarcoma is molecular testing, TLE1-targeted therapeutics could be used to selectively treat synovial sarcoma without damaging host tissue and could be a useful immunohistochemical marker in combination with CD37, CK7, EMA, BCL2, and MIC2 [27]. However, Kemal et al. have suggested that TLE1 expression is not specific to synovial sarcoma, based on their study of 163 soft tissue and bone neoplasms [29].

## TLE1 AND LUNG CANCER

TLE1 knockout mice exhibited lung hypoplasia and decreased OS. Additionally, TLE1 deficiency resulted in enhanced tumor growth [8]. TLE1 was found to be overexpressed in approximately 11% of patients with lung squamous cell carcinomas and 20% of patients with lung adenocarcinomas. It is a putative lung-specific oncogene that positively regulates Bcl2 expression and ErbB1/ErbB2 signaling to promote cancer progression [30].Interestingly, Yao et al. demonstrated that TLE1 promoted epithelial-to-mesenchymal transition in A549 human lung adenocarcinoma cells through transcriptional silencing of E-cadherin [31]. These results are consistent with experiments in Grg1 transgenic mice in which treatment with trichostatin A (TSA), a histone deacetylase (HDAC) inhibitor, inhibited lung tumorigenesis. Although TSA did not affect the total levels of Grg1in A549 cells, it reduced ErbB1 and ErbB2 expression. Therefore, HDAC inhibitors have therapeutic potential in lung cancer [32].

## DISTINCT FUNCTIONS OF TLE1 IN BREAST CANCER

The functions of TLE1 differ in breast compared to other cancers. Brunquell et al. discovered that TLE1 suppressed Bit1-mediated anoikis, which may be a novel mechanism by which the TLE1 transcriptional machinery is turned off, and induction of Bit1-mediated anoikis may be an effective treatment strategy for breast cancer. Importantly, TLE1 is selectively over-expressed in invasive breast tumors compared to non-invasive ductal carcinoma in situ and normal mammary epithelial tissue [33]. TLE1 is also critical for the binding of thee strogen receptor to chromatin [34]. Collectively, the data suggest TLE1 may be a novel target for breast cancer therapeutics.

## TLE1 AND HEMATOLOGIC MALIGNANCIES

Interestingly, in hematologic malignancies such as diffuse large B-cell lymphoma and acute myeloid leukemia, reintroduction of TLE1 into hypermethylated leukemia or lymphoma cells resulted in growth inhibition *in vitro* and *in vivo*. Conversely, depletion of TLE1 in unmethylated cells by shRNA enhanced tumor growth, indicating epigenetic inactivation of TLE1 promoted the development of hematologic malignancies by disrupting cell differentiation and growth-suppressive pathways [35]. The TLE1 gene functions as a tumor suppressor in myeloid leukemia. Epigenetic inactivation of the TLE1 gene was shown to promote myeloid cell proliferation and survival [36]. TLE1 acts as a repressor of AML1,which may regulate hematopoietic cell differentiation and proliferation. TLE1 binds to the Runt domain and the C-terminus of AML1 including the VWRPY motif [37]. TLE family proteins are regulated in part by amino-terminal enhancer of split (AES) expression, and are critical for hematopoietic cell self-renewal. Therefore, AES and the TLE family proteins are possible therapeutic targets [38]. Inhibition of TLE1 function may prevent cancer cell self-renewal resulting in decreased tumor burden. TLE1 in combination with LCP2, TNFRSF9, FUT8, and IRF4 could also be used for molecular subtyping in diffuse large B-cell lymphoma [39].

## TLE1 AND GLIOBLASTOMA

TLE1 plays a key role in neurogenesis. It can inhibit the differentiation of neural progenitor cells into neurons and is expressed in the postnatal brain. There is also evidence that by acting together with FOXG1, TLE1 can promote neuronal survival in a CK2- and PI3K-Akt-dependent manner [40]. Elevated expression of FOXG1 and TLE1 is frequently observed in glioblastoma (GBM), and is associated with poor overall survival. Intriguingly, inhibition of FOXG1 and TLE1 reduced the growth of brain tumor-initiating cells (BTICs), indicating that both proteins regulate glioblastoma development and progression [41]. This paper is the first to address the relationship between TLE1 and glioblastoma and gives us new hope in the fight against GBM. The next steps are to find the genes controlled by the two proteins and to identify other important regulators. All of these proteins or/and regulators may provide new potential therapeutic targets to reduce the tumorigenic ability of BTIs.

## TLE1 AND GASTRIC CANCER

Although TLE1 is not expressed in normal gastric mucosa, it has been detected in approximately 50% of gastric cancer patients, which is consistent with published data for breast and lung cancer [30, 33]. TLE1 expression was also associated with prognostic clinicopathological parameters, including gender, American Joint Committee on Cancer Stage, and tumor depth. These data indicate that TLE1 expression is a good prognostic indicator in gastric cancer and that it exerts oncogenic effects [42]. Unfortunately, the mechanism of TLE1 action in this cancer remains unknown.

## TLE1 AND HEPATOCELLULAR CARCINOMA

Early in 2010, a study showed that in four liver cancer cell lines (HuH6, Hep3B, HepG2, and HLE) down-regulation of TLE1 promoted tumorigenesis, suggesting that TLE1 may act as a tumor suppressor in hepatocellular carcinoma [43]. Three years later, Zhang et al. found that MicroRNA-657 promotes tumorigenesis in hepatocellular carcinoma by targeting TLE1 through NFkB pathways [44], which confirms the functions of TLE1 in hepatocellular carcinoma and provides new insight into the potential molecular mechanisms of hepatic carcinogenesis.

## ROLES OF OTHER TLE FAMILY MEMBERS IN CANCER

No studies have demonstrated a relationship between TLE1 and prostate cancer. However, expression of TLE family members (e.g. TLE3, an analog of TLE1) correlated with the degree of tumor differentiation. Interestingly, a new TLE3 isoform was identified that may be involved in prostate cancer development and progression [45]. Recent studies also suggest that TLE2 is specifically expressed in grade I astrocytoma as compared to normal tissue and aggressive astrocytoma [46]. It was also shown that TLE2 and TLE4 were over-expressed in pituitary adenomas [47], and that TLE4 promoted cell proliferation and invasion, in part via activation of a JNK-c-Jun pathway, and subsequently increased cyclinD1 and decreased P27Kip1 expression [48]. Although these studies are just solitary results with little impact on clinical therapies, they suggest that TLE family proteins are highly important in several tumors and warrant further investigation.

## TLE1 FUNCTION IN PANCREATIC CANCER

Many studies have demonstrated a relationship between TLE1 and various types of cancer (e.g. synovial sarcomas and lung cancer). Indeed, the data suggest that TLE1 is widely expressed in all types of cancer. However, the mechanisms underlying TLE1 function in pancreatic cancer have not yet been elucidated. Additionally, the expression of TLE1 has not been confirmed in pancreatic cancer. Quantitative reverse transcription PCR experiments have demonstrated that TLE1-4 is dynamically expressed during pancreas development [49]. Metzger et al. found that the transcriptional co-repressor Grg3/TLE3 promoted pancreatic endocrine progenitor delamination and β-cell differentiation [50]. More recently, they demonstrated that Grg3/TLE3 and Grg1/TLE1 induced monohormonal pancreatic β-cells while it repressed α-cell functions. Mouse Grg3 and human Grg1 can be used as markers of proper monohormonal β-cell differentiation *in vitro* [51]. Thus, additional studies are required to analyze the functions of TLE1 and other TLE family members in pancreatic cancer. An understanding of the molecular mechanisms underlying TLE1 function is required in order to develop novel targeted therapies for pancreatic cancer and prolong patient survival.

## CONCLUSIONS AND FUTURE PERSPECTIVES

TLE1 and related proteins have critical roles during development. For example, knockout of TLE3/4 inhibited embryonic stem cell differentiation [52]. Additionally, TLE proteins can function both directly and indirectly in tumorigenesis [53]. The expression of TLE family members can be used as diagnostic markers in several types of cancer including synovial sarcoma and malignant mesothelioma.

Studies of TLE1 structure and function indicate that it directly interacts with the N-terminus of histone H3 to promote chromatin remodeling. It prevents recruitment of transcriptional activators to repress transcription. The the functions of TLE1 in the Wnt, Notch, and other key signaling pathways have not been elucidated. Because TLE family proteins regulate transcription, the functions of TLE1 in pancreatic cancer should be investigated. We hypothesize that TLE1 expression maybe correlated with prognostic factors including T/N stage and overall survival.

TLE proteins are relatively challenging drug targets because they lack an enzymatic active site. Therefore, factors upstream and/or downstream of TLE proteins should also be considered as therapeutic targets. Many TLE-interacting proteins act as transcriptional activators or repressors in tumor-associated signaling pathways. TLE1 may help maintain the equilibrium between transcriptional activation and repression. In addition, TLE1 regulates the NF-κB inflammatory pathway and the immune response, which can also affect the immune microenvironment [8]. In summary, mechanistic studies of TLE1 function in pancreatic and other cancers are required in order to develop new targeted therapeutics for cancer.

## References

[1] Torre LA, Bray F, Siegel RL, Ferlay J, Lortet-Tieulent J, Jemal A (2015). Global cancer statistics, 2012. CA Cancer J Clin.

[2] Ferlay J, Soerjomataram I, Ervik M, Dikshit R, Eser S, Mathers C, Rebelo M, Parkin DM, Forman D, Bray F, Shin HR, Steliarova-Foucher E (2012). GLOBOCAN 2012. Cancer Incidence and Mortality Worldwide.

[3] Siegel RL, Miller KD, Jemal A (2016). Cancer statistics, 2016. CA Cancer J Clin.

[4] Rahib L, Smith BD, Aizenberg R, Rosenzweig AB, Fleshman JM, Matrisian LM (2014). Projecting cancer incidence and deaths to 2030: the unexpected burden of thyroid, liver, and pancreas cancers in the United States. Cancer Res.

[5] Chen W, Zheng R, Zuo T, Zeng H, Zhang S, He J (2016). National cancer incidence and mortality in China, 2012. Chin J Cancer Res.

[6] Jones S, Zhang X, Parsons DW, Lin JC, Leary RJ, Angenendt P, Mankoo P, Carter H, Kamiyama H, Jimeno A, Hong SM, Fu B, Lin MT, Calhoun ES, Kamiyama M, Walter K (2008). Core signaling pathways in human pancreatic cancers revealed by global genomic analyses. Science.

[7] Kaul A, Schuster E, Jennings BH (2014). The Groucho co-repressor is primarily recruited to local target sites in active chromatin to attenuate transcription. PLoS Genet.

[8] Ramasamy S, Saez B, Mukhopadhyay S, Ding D, Ahmed AM, Chen X, Pucci F, Yamin R, Wang J, Pittet MJ, Kelleher CM, Scadden DT, Sweetser DA (2016). Tle1 tumor suppressor negatively regulates inflammation in vivo and modulates NF-kappaB inflammatory pathway.

[9] Liu Y, Dehni G, Purcell KJ, Sokolow J, Carcangiu ML, Artavanis-Tsakonas S, Stifani S (1996). Epithelial expression and chromosomal location of human TLE genes: implications for notch signaling and neoplasia. Genomics.

[10] Jennings BH, Ish-Horowicz D (2008). The Groucho/TLE/Grg family of transcriptional co-repressors. Genome Biol.

[11] Patel SR, Bhumbra SS, Paknikar RS, Dressler GR (2012). Epigenetic mechanisms of Groucho/Grg/TLE mediated transcriptional repression. Molecular cell.

[12] Stifani S, Blaumueller CM, Redhead NJ, Hill RE, Artavanis-Tsakonas S (1992). Human homologs of a Drosophila Enhancer of split gene product define a novel family of nuclear proteins. Nat Genet.

[13] Gasperowicz M, Otto F (2005). Mammalian Groucho homologs: redundancy or specificity?. J Cell Biochem.

[14] Palaparti A, Baratz A, Stifani S (1997). The Groucho/transducin-like enhancer of split transcriptional repressors interact with the genetically defined amino-terminal silencing domain of histone H3. J Biol Chem.

[15] Dasen JS, JP Martinez Barbera, Herman TS, Connell SO, Olson L, Ju B, Tollkuhn J, Baek SH, Rose DW, Rosenfeld MG (2001). Temporal regulation of a paired-like homeodomain repressor/TLE corepressor complex and a related activator is required for pituitary organogenesis. Genes & development.

[16] Wang W, Wang YG, Reginato AM, Glotzer DJ, Fukai N, Plotkina S, Karsenty G, Olsen BR (2004). Groucho homologue Grg5 interacts with the transcription factor Runx2-Cbfa1 and modulates its activity during postnatal growth in mice. Developmental biology.

[17] Muhr J, Andersson E, Persson M, Jessell TM, Ericson J (2001). Groucho-mediated transcriptional repression establishes progenitor cell pattern and neuronal fate in the ventral neural tube. Cell.

[18] Agarwal M, Kumar P, Mathew SJ (2015). The Groucho/Transducin-like enhancer of split protein family in animal development. IUBMB life.

[19] Palaparti A, Baratz A, S S (1997). The Groucho/transducin-like enhancer of split transcriptional repressors interact with the genetically defined amino-terminal silencing domain of histone H3. J Biol Chem.

[20] Kaul AK, Schuster EF, Jennings BH (2015). Recent insights into Groucho co-repressor recruitment and function. Transcription.

[21] Jan Y, Matter M, Pai JT, Chen YL, Pilch J, Komatsu M, Ong E, Fukuda M, Ruoslahti E (2004). A mitochondrial protein, Bit1, mediates apoptosis regulated by integrins and Groucho/TLE corepressors. Cell.

[22] Terry J, Saito T, Subramanian S, Ruttan C, Antonescu CR, Goldblum JR, Downs-Kelly E, Corless CL, Rubin BP, van de Rijn M, Ladanyi M, Nielsen TO (2007). TLE1 as a diagnostic immunohistochemical marker for synovial sarcoma emerging from gene expression profiling studies. The American journal of surgical pathology.

[23] Foo WC, Cruise MW, Wick MR, Hornick JL (2011). Immunohistochemical staining for TLE1 distinguishes synovial sarcoma from histologic mimics. American journal of clinical pathology.

[24] Valente AL, Tull J, Zhang S (2013). Specificity of TLE1 expression in unclassified high-grade sarcomas for the diagnosis of synovial sarcoma. Applied immunohistochemistry & molecular morphology.

[25] Chuang HC, Hsu SC, Huang CG, Hsueh S, Ng KF, Chen TC (2013). Reappraisal of TLE-1 immunohistochemical staining and molecular detection of SS18-SSX fusion transcripts for synovial sarcoma. Pathology international.

[26] Atef A, Alrashidy M (2015). Transducer-like Enhancer of Split 1 as a Novel Immunohistochemical Marker for Diagnosis of Synovial Sarcoma. Asian Pacific Journal of Cancer Prevention.

[27] Rekhi B, Basak R, Desai SB, Jambhekar NA (2012). Immunohistochemical validation of TLE1, a novel marker, for synovial sarcomas. Indian J Med Res.

[28] Seo SW, Lee H, Lee HI, Kim HS (2011). The role of TLE1 in synovial sarcoma. Journal of orthopaedic research.

[29] Kosemehmetoglu K, Vrana JA, Folpe AL (2009). TLE1 expression is not specific for synovial sarcoma: a whole section study of 163 soft tissue and bone neoplasms. Modern pathology.

[30] Allen T, Van Tuyl M, Iyengar P, Jothy S, Post M, Tsao MS, Lobe CG (2006). Grg1 acts as a lung-specific oncogene in a transgenic mouse model. Cancer Res.

[31] Yao X, Ireland SK, Pham T, Temple B, Chen R, Raj MH, Biliran H (2014). TLE1 promotes EMT in A549 lung cancer cells through suppression of E-cadherin. Biochemical and biophysical research communications.

[32] Liu J, Li Y, Dong F, Li L, Masuda T, Allen TD, Lobe CG (2015). Trichostatin A suppresses lung adenocarcinoma development in Grg1 overexpressing transgenic mice. Biochemical and biophysical research communications.

[33] Brunquell C, Biliran H, Jennings S, Ireland SK, Chen R, Ruoslahti E (2012). TLE1 is an anoikis regulator and is downregulated by Bit1 in breast cancer cells. Molecular cancer research : MCR.

[34] Holmes KA, Hurtado A, Brown GD, Launchbury R, Ross-Innes CS, Hadfield J, Odom DT, Carroll JS (2012). Transducin-like enhancer protein 1 mediates estrogen receptor binding and transcriptional activity in breast cancer cells.

[35] Fraga MF, Berdasco M, Ballestar E, Ropero S, Lopez-Nieva P, Lopez-Serra L, Martin-Subero JI, Calasanz MJ, I Lopez de Silanes, Setien F, Casado S, Fernandez AF, Siebert R, Stifani S, Esteller M (2008). Epigenetic inactivation of the Groucho homologue gene TLE1 in hematologic malignancies. Cancer Res.

[36] Dayyani F, Wang J, Yeh JR, Ahn EY, Tobey E, Zhang DE, Bernstein ID, Peterson RT, Sweetser DA (2008). Loss of TLE1 and TLE4 from the del(9q) commonly deleted region in AML cooperates with AML1-ETO to affect myeloid cell proliferation and survival. Blood.

[37] Imai Y, Kurokawa M, Tanaka K, Friedman AD, Ogawa S, Mitani K, Yazaki Y, Hirai H (1998). TLE, the human homolog of groucho, interacts with AML1 and acts as a repressor of AML1-induced transactivation. Biochemical and biophysical research communications.

[38] Steffen B, Knop M, Bergholz U, Vakhrusheva O, Rode M, Kohler G, Henrichs MP, Bulk E, Hehn S, Stehling M, Dugas M, Baumer N, Tschanter P, Brandts C, Koschmieder S, Berdel WE (2011). AML1/ETO induces self-renewal in hematopoietic progenitor cells via the Groucho-related amino-terminal AES protein. Blood.

[39] Li C, Zhu B, Chen J, Huang X (2015). Novel prognostic genes of diffuse large B-cell lymphoma revealed by survival analysis of gene expression data. OncoTargets and therapy.

[40] Dastidar SG, Narayanan S, Stifani S, D’Mello SR (2012). Transducin-like enhancer of Split-1 (TLE1) combines with Forkhead box protein G1 (FoxG1) to promote neuronal survival. J Biol Chem.

[41] Verginelli F, Perin A, Dali R, Fung KH, Lo R, Longatti P, Guiot MC, RF Del Maestro, Rossi S, di Porzio U, Stechishin O, Weiss S, Stifani S (2013). Transcription factors FOXG1 and Groucho/TLE promote glioblastoma growth. Nature communications.

[42] Lee JH, Son MW, Kim KJ, Oh MH, Cho H, Lee HJ, Jang SH, Lee MS (2016). Prognostic and Clinicopathological Significance of Transducer-Like Enhancer of Split 1 Expression in Gastric Cancer. Journal of gastric cancer.

[43] Di Masi A, Viganotti M, Antoccia A, Magrelli A, Salvatore M, Azzalin G, Tosto F, Lorenzetti S, Maranghi F, Mantovani A, Macino G, Tanzarella C, Taruscio D (2010). Characterization of HUH6, HEP3B, HEPG2 and HLE liver cancer cell lines by WNT/Q- catenin pathway, microrna expression and protein expression profile. Cell Mol Biol (Noisy-le-grand).

[44] Zhang L, Yang L, Liu X, Chen W, Chang L, Chen L, Loera S, Chu P, Huang WC, Liu YR, Yen Y (2013). MicroRNA-657 promotes tumorigenesis in hepatocellular carcinoma by targeting transducin-like enhancer protein 1 through nuclear factor kappa B pathways. Hepatology (Baltimore, Md).

[45] Nakaya HI, Beckedorff FC, Baldini ML, Fachel AA, Reis EM, Verjovski-Almeida S (2007). Splice variants of TLE family genes and up-regulation of a TLE3 isoform in prostate tumors. Biochemical and biophysical research communications.

[46] Rorive S, Maris C, Debeir O, Sandras F, Vidaud M, Bieche I, Salmon I, Decaestecker C (2006). Exploring the distinctive biological characteristics of pilocytic and low-grade diffuse astrocytomas using microarray gene expression profiles. Journal of neuropathology and experimental neurology.

[47] Moreno CS, Evans CO, Zhan X, Okor M, Desiderio DM, Oyesiku NM (2005). Novel molecular signaling and classification of human clinically nonfunctional pituitary adenomas identified by gene expression profiling and proteomic analyses. Cancer Res.

[48] Wang SY, Gao K, Deng DL, Cai JJ, Xiao ZY, He LQ, Jiao HL, Ye YP, Yang RW, Li TT, Liang L, Liao WT, Ding YQ (2016). TLE4 promotes colorectal cancer progression through activation of JNK/c-Jun signaling pathway. Oncotarget.

[49] Hoffman BG, Zavaglia B, Beach M, Helgason CD (2008). Expression of Groucho/TLE proteins during pancreas development. BMC developmental biology.

[50] Metzger DE, Gasperowicz M, Otto F, Cross JC, Gradwohl G, Zaret KS (2012). The transcriptional co-repressor Grg3/Tle3 promotes pancreatic endocrine progenitor delamination and beta-cell differentiation. Development (Cambridge, England).

[51] Metzger DE, Liu C, Ziaie AS, Naji A, Zaret KS (2014). Grg3/TLE3 and Grg1/TLE1 induce monohormonal pancreatic beta-cells while repressing alpha-cell functions. Diabetes.

[52] Laing AF, Lowell S, Brickman JM (2015). Gro/TLE enables embryonic stem cell differentiation by repressing pluripotent gene expression. Developmental biology.

[53] Buscarlet M, Stifani S (2007). The ‘Marx’ of Groucho on development and disease. Trends Cell Biol.

